# Potentiation of Antibiotic Action and Efflux Pump Inhibitory Effect on *Staphylococcus aureus* Strains by Solasodine

**DOI:** 10.3390/antibiotics11101309

**Published:** 2022-09-27

**Authors:** Ana Raquel Pereira da Silva, Maria do Socorro Costa, Nara Juliana Santos Araújo, Thiago Sampaio de Freitas, Ray Silva de Almeida, José Maria Barbosa Filho, Josean Fechine Tavares, Erlânio Oliveira de Souza, Pablo Antonio Maia de Farias, Jacqueline Cosmo Andrade Pinheiro, Henrique Douglas Melo Coutinho

**Affiliations:** 1 Graduate Program in Biotechnology, Universidade Estadual do Ceará—UECE, Fortaleza 60714-903, Brazil; 2 Graduate Program in Health Sciences, Universidade Federal do Cariri—UFCA, Barbalha 63180-000, Brazil; 3 Department of Biological Chemistry, Universidade Regional do Cariri—URCA, Crato 63105-000, Brazil; 4 Laboratory of Pharmaceutical Technology, Universidade Federal da Paraíba—UFPB, João Pessoa 58033-455, Brazil; 5 Department of Pharmaceutical Sciences, Universidade Federal da Paraíba—UFPB, João Pessoa 58051-900, Brazil; 6 Faculty of Technology of Cariri, Juazeiro do Norte 63040-000, Brazil; 7 College of Dentistry—CECAPE, Juazeiro do Norte 63024-015, Brazil

**Keywords:** antibacterial activity, bacterial resistance, sapogenin

## Abstract

A worrisome fact is the increase in microbial resistance, which has as its main cause the indiscriminate use of antibiotics. Scientific studies have investigated bioactive compounds such as steroidal sapogenins, in the perspective of new beneficial alternatives for the control of bacterial resistance. Therefore, the objective of this work was to verify the antibacterial activity as well as the modifying action of antibiotics associated with solasodine and its ability to inhibit the efflux pump mechanism in strains of *Staphylococcus aureus*. Tests were performed to verify the minimum inhibitory concentration (MIC). In addition, the action-modifying potential of antibiotics and the inhibitory capacity of the efflux pump NorA and MepA through synergistic effects on the antibiotic and ethidium bromide were evaluated. Solasodine showed significant results for the standard bacteria with an MIC of 512 μg/mL, and when associated with the antibiotics gentamicin and nofloxacin for the multidrug-resistant bacteria *S. aureus* 10, *Escherichia coli* 06, and *Pseudomonas aeruginosa* 24, it showed a 50% reduction in MIC. The association of solasodine with the antibiotic ciprofloxacin against *S. aureus* K2068 (MepA) showed synergism, with a reduction in the MIC of the antibiotic from 64 μg/mL to 40 μg/mL, and also a reduction in the MIC when the antibiotic was used in conjunction with the efflux pump inhibitors. Solasodine may be acting on the mechanism of action of the antibiotic, as it has shown a potentiating effect when associated with antibiotics, inducing a reduction in the MIC against Gram-positive and Gram-negative bacteria. Therefore, this study demonstrated significant results for the potentiating action of solasodine when associated with antibiotics of clinical importance.

## 1. Introduction

Antibiotics are drugs that have revolutionized the treatment of diseases caused by bacteria. However, infectious diseases are currently one of the leading causes of death in the world, due to the increasing number of microorganisms that are less susceptible to drugs, thus resulting in a worrying increase in acquired bacterial resistance, either by mechanisms of mutation, transformation, or conjunction; these processes of bacterial adaptation that were supposed to be natural have resulted in a consequent phenomenon of resistance, characterized as a serious health problem that has as its main cause the indiscriminate use of antibiotics that consequently causes a succession of adverse events to the human microbiota [1,2,3].

The mechanism of action, such as the efflux pump present in some bacteria, is one of the causes of antibiotic resistance, since it promotes active pumping of antimicrobials from the intracellular to the extracellular environment, in which its active flux produces resistance to certain antimicrobials [4]. NorA is a multidrug efflux pump and one of the most studied systems; it is a pump present in the bacterium *Staphylococcus aureus* [5]. MepA is another multidrug efflux pump belonging to the multidrug and toxic compound extrusion (MATE) family also present in *S. aureus* [6].

Therefore, antimicrobial resistance is an issue of worldwide concern and scientific studies have investigated natural products as a source of new drugs [7], and affordable and beneficial alternatives that can contribute to the control of microbial resistance. They have also investigated the use of antibiotics of conventional use associated with bioactive compounds of biotechnological interest, in the perspective of a new potentiating agent of the effect of drugs, contributing in the alteration of the permeability of the cellular membrane of the bacteria or a possible inhibiting effect of the efflux pump mechanism [8,9].

Steroidal compounds have been valuable precursors to complementary sources for the synthesis of various drugs. Sapogenins are already known for their multiple structural variations and pharmacological properties that can be used in industry, both in human and veterinary medicinal applications [10,11,12]. 

Solasodine is a steroidal alkaloid, found in nature mainly in plants of the genus *Solanum*, being widely studied for its pharmacological applications in view of the presence of steroidal sapogenins that have pharmaceutical and industrial importance [11,13,14]. Thus, the interest in promoting this work is due to the activities already described in previous studies, in which solasodine demonstrates anticonvulsant [15], anti-inflammatory [16], antioxidant [17], antiviral [18], antifungal [19,20], anticancer [21,22,23], and antibacterial activity [24,25,26].

This study aimed to verify the antibacterial activity and the modifying action of antibiotics by the broth microdilution method, as well as the ability for solasodine to inhibit the NorA and MepA efflux pump mechanism.

## 2. Results

### 2.1. Antibacterial Activity

With the minimum inhibitory concentration (MIC) analysis, solasodine was found to have no direct antibacterial activity for the bacteria *S. aureus* 10, *S. aureus* K2068, *P. aeruginosa* 24, and *E. coli* 06, showing an MIC greater than or equal to 1024 μg/mL.

However, for the bacteria *S. aureus* 1199B, *S. aureus* ATCC 25923, *P. aeruginosa* ATCC 9027, and *E. coli* ATCC 25922, solasodine showed an MIC of 512 μg/mL, as presented in Table 1.

### 2.2. Antibiotic Activity Modifier Action

**Figure 1:**
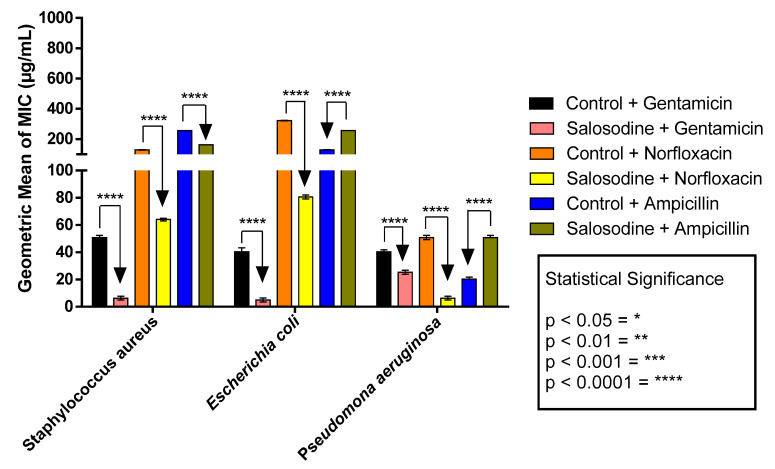
Modifying effect of antibiotics by solasodine against *S. aureus* 10, *E. coli* 06, and *P. aeruginosa* 24. The control refers to antibiotics. **** Statistically significant value when *p* < 0.0001; not statistically significant when *p* > 0.05.

Solasodine in association with gentamicin demonstrated an antibiotic potentiation effect against *S. aureus* 10 with MIC reduction from 50 μg/mL to 6 μg/mL, *Escherichia coli* 06 with MIC reduction from 40 μg/mL to 5 μg/mL, and *Pseudomonas aeruginosa* 24 with MIC reduction from 40 μg/mL to 25 μg/mL. The association of solasodine with norfloxacin also showed an antibiotic potentiation effect against *S. aureus* 10 with MIC reduction from 128 μg/mL to 64, *E. coli* 06 with MIC reduction from 322 μg/mL to 80 μg/mL, and *P. aeruginosa* 24 with MIC reduction from 50 μg/mL to 6 μg/mL (Figure 1).

For solasodine in association with ampicillin, antibiotic potentiation was only observed against *S. aureus* 10 with a reduction in the MIC from 256 μg/mL to 161 μg/mL. Against *E. coli* 06 and *P. aeruginosa* 24, the association of sapogenin with the antibiotic expressed itself in an antagonistic effect with an increase in the MIC (Figure 1).

### 2.3. Efflux Pump Inhibitory Effect Nora E Mepa

**Figure 2:**
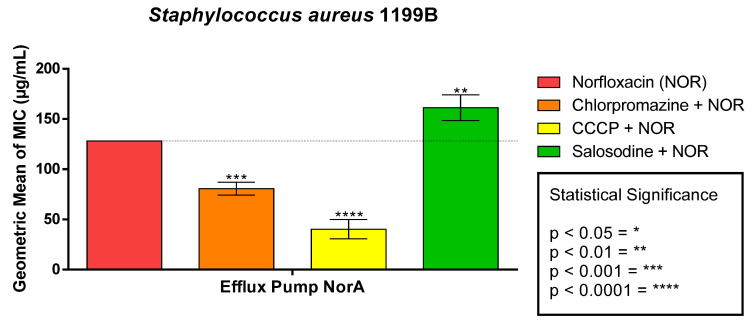
Modifying effect of norfloxacin antibiotic action by solasodine on *S. aureus* strain 1199B.

The association of solasodine with the antibiotic norfloxacin against *S. aureus* 1199B resulted in an antagonistic effect with an increase in the MIC. However, when norfloxacin was tested together with the efflux pump inhibitors chlorpromazine and CCCP, it showed a reduction of the MIC (Figure 2).

**Figure 3:**
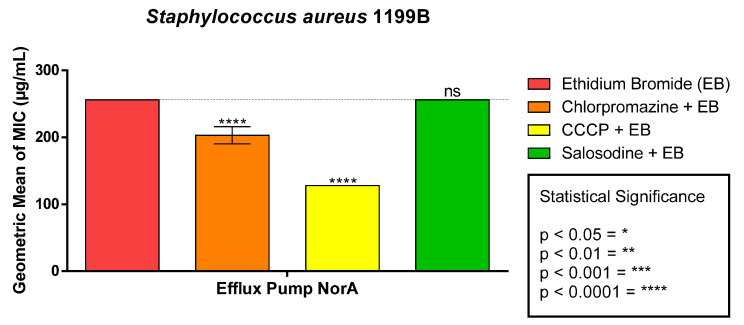
Modifying effect of ethidium bromide by solasodine on *S. aureus* strain 1199B.

When solasodine was associated with ethidium bromide, it did not show a statistically significant result. However, there was a reduction in the MIC of the bromide in conjunction with the efflux pump inhibitors (Figure 3).

**Figure 4:**
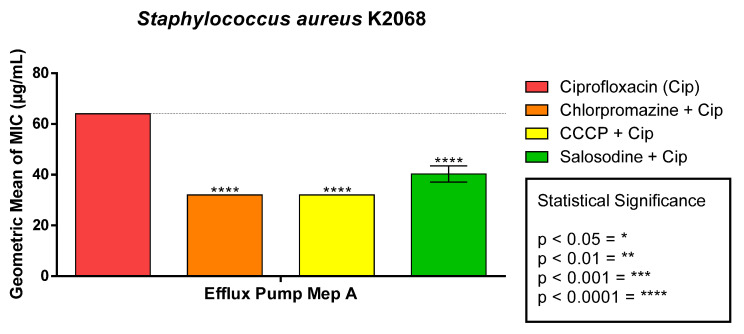
Modifying effect of norfloxacin antibiotic action by solasodine on *S. aureus* strain K2068.

The association of solasodine with the antibiotic ciprofloxacin against *S. aureus* K2068 demonstrated antibiotic potentiation with a reduction in the MIC from 64 μg/mL to 40 μg/mL. It also resulted in a reduction in the MIC of ciprofloxacin when tested in conjunction with the efflux pump inhibitors (Figure 4). This suggests that solasodine may be acting on the mechanism of action of the antibiotic, increasing its effect against the bacteria tested.

**Figure 5:**
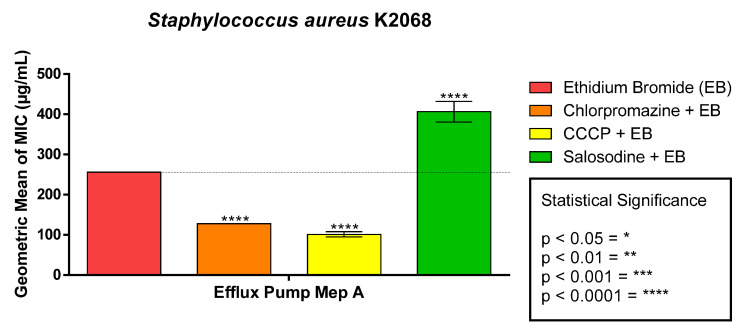
Modifying effect of ethidium bromide by solasodine on *S. aureus* strain K206B.

The solasodine associated with ethidium bromide expressed an antagonistic effect, having a high increase in the MIC. The bromide in conjunction with the standard inhibitors showed a reduction in the MIC (Figure 5). 

## 3. Discussion 

Bacteria have the ability to adapt easily to the environment, in which they can develop several complex mechanisms, such as altering membrane permeability, producing enzymes capable of inactivating the action of antibiotics, and modifying the molecular structure; these factors cause resistance [27]. However, rather than replacing antibiotics that are ineffective for MDR bacteria, combining them with bioactive compounds is a new alternative for antimicrobial therapy. The combination of steroidal alkaloids with antibiotics can contribute to bacterial inhibition and the restoration of antibiotic efficacy [28,29], since the main antibacterial mechanisms of alkaloids include alteration in cell membrane permeability, inhibition of bacterial cell wall synthesis, inhibition of bacterial metabolism, efflux pump inhibition, and inhibition of nucleic acid and protein synthesis [30,31]. 

The analysis of the antibacterial activity of solasodine demonstrated a reduction in the MIC for the standard bacteria *S. aureus*, *P. aeruginosa*, and *E. coli*. This result is in agreement with the antibacterial effect reported by [24,25], where solasodine is shown to have an effect against Gram-positive and Gram-negative bacteria. Therefore, the intrinsic activity of solasodine may be related to the interaction of this alkaloid with the bacterial cell wall structure.

Niño et al. [25] evaluated steroidal alkaloids isolated from *Solanum* and reported that the antibacterial activity of solasodine against *S. aureus* is probably through the inhibition of DNA topoisomerase II; this may be attributed to the fact that alkaloids possess mechanisms for inhibiting bacterial nucleic acid and protein synthesis [31]. In addition, alkaloids and steroids present in other plant species are also associated with antimicrobial activity against Gram-positive bacteria [32], which has a thicker peptidoglycan layer in its cell wall, and the synthesis of this layer involves the participation of a beta-lactam antibiotic [33].

Bibon [26] evaluated solasodine isolated from a species of the genus *Solanum* against *E. coli*; he classified the antibacterial efficacy of this sapogenin as lethal, due to solasodine damaging the DNA of the bacteria, proving that solasodine indeed presents a damaging activity to the bacterial DNA. This is in agreement with the results of this study, in which the Gram-negative bacteria *E. coli* 06 and *P. aeruginosa* 24 also showed significant results. 

In the antibiotic potentiation activity where solasodine was associated with antibiotics from the aminoglycoside group, flouroquinolones and a beta-lactam, a reduction in the MIC for the three MDR bacteria tested was shown. The solasodine may be acting to increase the effect of these antibiotics against the strains tested. This effect may be alternatively related to synergism, which is a positive interaction of a compound when associated with an antibiotic, resulting in a reduction in the MIC of the antibiotic used, as well as a decrease in the appearance of resistant bacterial cells [34]. It can also be attributed to the fact that the solasodine alkaloid contributes to the alteration of cell membrane permeability with the uptake of molecules through porin channels [27,31].

The antagonistic effect was also presented by the association of solasodine with the antibiotics ampicillin and norfloxacin, having resulted in the decrease of the effect of both combinations. It can be explained by the fact that synergistic and antagonistic interactions are dependent on the mechanism of bacterial inhibition by the class of antibiotic [34], as well as the possibility of an action arising from the chelation mechanism of the antibiotic when associated with solasodine against the bacteria tested [8]. Additionally, the antagonism often coincides with the increase of ethidium bromide of the bacteria [35].

Therefore, for this work, the MIC of ethidium bromide showed a reduction in conjunction with the inhibitors, thus demonstrating that the bacteria tested have an active efflux pump mechanism from the intracellular to the extracellular medium [36].

## 4. Materials and Methods

### 4.1. Substances, Antibiotics and Reagents

**Figure 6:**
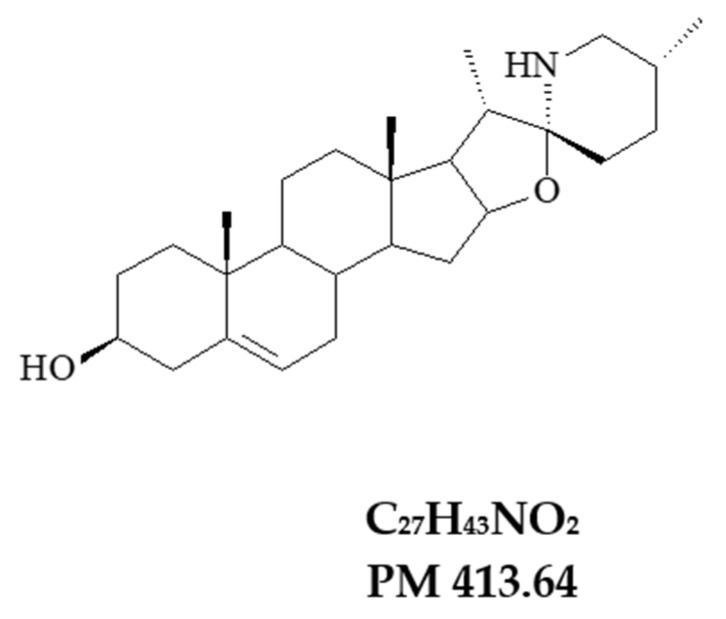
Solasodine.

Salosodine (Figure 6) was obtained from the species *Solanum paludosum* Moric., was first dissolved in dimethyl sulfoxide (DMSO) and then diluted in sterile water to obtain the concentrations of (1024 μg/mL), (512 μg/ML) and (128 μg/mL).

In the test to evaluate the action-modifying activity of antibiotics, gentamicin, norfloxacin, and ampicillin (Sigma Aldrich Co. Ltd., São Paulo, Brazil) were used at a concentration of (1024 μg/mL).

In the efflux pump inhibitory effect test, the following were used: chlorpromazine (CPZ), carbonyl-m-chlorophenyl hydrazone cyanide (CCCP), and ethidium bromide (Sigma Aldrich Co. Ltd., São Paulo, Brazil) at a concentration of (1024 μg/mL). Norfloxacin and ciprofloxacin antibiotics specific for NorA and MepA were also used at a concentration of (1024 μg/mL). Ethidium bromide and the antibiotics were diluted in sterile water, except for norfloxacin and CPZ which followed the same dilution process as solasodine. CCCP was dissolved in a 1:1 methanol/water solution to obtain the concentration (1024 μg/mL).

### 4.2. Bacterial Strains

In the test to verify antibacterial activity, the standard strains used were *Escherichia coli* ATCC 25922, *Staphylococcus aureus* ATCC 25923, *Pseudomonas aeruginosa* ATCC 902, and the multidrug-resistant strains *Escherichia coli* 06, *Staphylococcus aureus* 10, *Pseudomonas aeruginosa* 24, obtained through the Microbiology and Molecular Biology Laboratory (LMBM) University of Region of Cariri (URCA, Crato, Brazil). Before performing each test, they were cultured for 24 h at 37 °C in Brain Heart infusion agar (BHI) (Laboratorios cond S.A., Madrid, Spain).

*Staphylococcus aureus* strains SA-1199B (NorA overexpressed) and SA-K2068 (MepA expressed), provided by Prof. S. Gibbons (University of London, London, England), were used to test the inhibitory effect of the efflux pump. Initially, they were maintained in blood agar (Laboratory Difco Ltda., Franklin Lakes, NJ, USA), but before testing they were cultured for 24 h at 37 °C in Brain Heart infusion agar (BHI) (Laboratorios cond S.A., Madrid, Spain). All culture media were prepared according to the manufacturer’s instructions.

### 4.3. Determination of the Minimum Inhibitory Concentration (MIC)

To determine the minimum inhibitory concentration, the methodology used was CLSI [37] with modifications. Suspensions of the microorganisms were prepared in tubes containing 4 mL of sterile solution (NaCl a 0.9 %). These suspensions were then stirred with the aid of a vortex apparatus and the turbidity was compared and adjusted to that presented by the barium sulfate suspension in the 0.5 tube of the McFarland scale, which corresponds to an inoculum of approximately 10^5^ colony forming units/mL (CFU/mL). A solution containing 900 μL of 10% BHI broth and 100 μL of the inoculum was prepared in each eppendorf, and 100 μL of the solution was dispensed into each well of the 96-well microdilution plate. We also added 100 μL of solasodine at the initial concentration of (1024 μg/mL) and it was passed to the other wells through successive dilutions in a 1:1 ratio, ranging from (512 μg/mL) in the first well to (4 μg/mL) in the penultimate well, with the last well reserved for the test control.

To analyze the MIC, after incubating the plates for 24 h at 37 °C, an indicator solution of sodium resazurin in sterile distilled water at a concentration of 0.01% was prepared and 20 μL of this indicator solution was added to each well and the plates were incubated for 1 hour at room temperature. Color change from blue to pink was identified as proof of bacterial growth. The MIC is defined as the lowest concentration capable of inhibiting bacterial growth, which was evidenced by the unchanged blue color.

### 4.4. Evaluation of Modifying Antibiotic Activity Action

For the antibiotic action modifier activity, solasodine was tested at sub-inhibitory concentrations (MIC/8); the methodology proposed by Coutinho et al. [38] was used.

For the test, eppendorfs containing the solasodine at a concentration of (128 μg/mL) were prepared and suspensions of 10^5^ CFU/mL of the microorganisms were deposited together with BHI medium. A control for the antibiotic was prepared with the same amount of bacterial inoculum corresponding to the 10% volume in the eppendorf and 1350 µL of BHI. We dispensed 100 μL of these solutions into the cavities of the 96-well plate. Then 100 μL of each antibiotic was added at a concentration of (1024 μg/mL) following with successive microdilutions at a 1:1 ratio, ranging from (512 μg/mL) in the first well to (0.5 μg/mL) in the penultimate well, with the last well reserved for the test control. The plates were incubated for 24 h at 37 °C and then read using the colorimetric indicator resazurin.

### 4.5. Evaluation of Efflux Pump Inhibitory Effect Nora E Mepa 

In the efflux pump inhibition test, solasodine was tested at sub-inhibitory concentrations (MIC/8). The inhibitors CPZ, CCCP, and ethidium bromide were used to verify the effect. The methodology used was Tintino et al. [39] with modifications.

For analysis of efflux pump inhibition, solasodine was tested at concentrations of 128 μg/mL and 64 μg/mL. Eppendorfs were prepared containing solasodine and the bacterial inoculum for each strain tested, and the final volume was filled with BHI medium. In preparing the control group, 150 µL of the bacterial inoculum was transferred to Eppendorfs and 1350 mL of BHI was added. We distributed 100 µL of these solutions in 96-well microplates following with successive dilutions at a 1:1 ratio, with 100 µL of the antibiotic corresponding to each strain, or ethidium bromide, both at an initial concentration of 1024 µg/mL, with final concentrations varying from 512 µg/mL to 0.5 µg/mL. After 24 h of incubation of the plates, the reading was performed using the colorimetric indicator for microbiological tests.

### 4.6. Statistical Analysis

The data were analyzed using the two-way ANOVA test, in which the results were expressed as geometric mean and standard deviation using the GraphPad Prisma software version 5.0. It was subjected to the Bonferrini post hoc test, and the results were considered significant when *p* < 0.05, *p* < 0.0001, and not significant when *p* > 0.05.

## 5. Conclusions 

In the evaluation of antibacterial activity by the broth microdilution method, solasodine showed no clinically relevant intrinsic activity for four of the multidrug-resistant bacteria tested, showing a reduction in MIC only for *S. aureus* 1199B. However, as a modifier for the action of antibiotics, significant results against Gram-positive and Gram-negative strains were identified for association with the antibiotics gentamicin and norfloxacin, having a reduction of MIC by 50%. For association with ampicillin, only *S. aureus* 10 showed synergism.

Despite not showing direct antibacterial activity, solasodine demonstrated a synergistic effect when associated with the ciprofloxacin, inducing a reduction in the minimum inhibitory concentration, indicating an MePA efflux pump inhibitory effect. 

Therefore, the data obtained in this study are significant and may stimulate future research on the use of sapogenins as associated compounds to enhance drug activity, thus reducing possible mechanisms of bacterial resistance to antibiotics.

## Figures and Tables

**Table 1:** Determination of the minimum inhibitory concentration (μg/mL).

Bacteria	S.A 10	P.A 24	E.C 06	S.A K2068	S.A 1199B	S.A ATCC 25923	P.A ATCC 9027	E.C ATCC 25922
**Solasodine**	≥1024	≥1024	≥1024	≥1024	512	512	512	512

Abbreviations: S.A: *Staphylococcus aureus*; P.A: *Pseudomonas aeruginosa*, E.C: *Escherichia coli*.
